# Pontine Tuberculoma Mimicking an Astrocytic Tumor: A Case Report

**DOI:** 10.7759/cureus.80881

**Published:** 2025-03-20

**Authors:** Fiacro Jimenez-Ponce, Noé Pérez Carrillo, Jesús Q. Beltrán, Ylián Ramírez Tapia, Jose D Carrillo-Ruiz, Erick Gomez Apo, Cesar A Durán-López, José Luis Navarro-Olvera

**Affiliations:** 1 Neurosurgery, Hospital General de México "Dr. Eduardo Liceaga", Mexico City, MEX; 2 Research, Hospital Angeles Pedregal, Mexico City, MEX; 3 Anesthesiology, Hospital General de México "Dr. Eduardo Liceaga", Mexico City, MEX; 4 Functional Neurosurgery, General Hospital of Mexico, Mexico City, MEX; 5 Neuroscience, Anáhuac University, Mexico City, MEX; 6 Neuropathology, Hospital General de México "Dr. Eduardo Liceaga", Mexico City, MEX; 7 Stereotactic and Functional Neurosurgery, General Hospital of Mexico, Mexico City, MEX

**Keywords:** astrocytic tumor, neurosurgery, pons, surgical case report, tuberculoma

## Abstract

Tuberculomas located in the brainstem are rare entities. This article presents a case study detailing the diagnosis and treatment process of a patient with a pontine tuberculoma, as well as a literature review of tuberculomas located in the pons. A 39-year-old male patient showed progressive quadriparesis and deficits in VII, XI, and XII cranial nerves. MRI image initially suggested a pontine astrocytoma. A second biopsy revealed the presence of acid-fast bacilli. Following six months of anti-tuberculous treatment, the patient showed successful recovery.

## Introduction

Tuberculosis (TB) in the central nervous system is relatively common in developing countries with limited sources. The incidence ranks between 0.15 and 0.18% [1]. Tuberculomas in brain stem represent 5% of all intracranial tuberculomas in endemic zones [2]. Tuberculomas in the central nervous system can occur in both immunocompromised and healthy patients. Additionally, physicians should attend to solitary tuberculoma lesions that could be misdiagnosed as neoplasia. There are various clinical and imaging features associated with a narrow spectrum of neuroanatomical syndromes. Magnetic resonance imaging (MRI) is a useful tool for diagnosis. The tuberculomas are isointense or hypointense on the T1-weighted sequence; on the T2, the hypointense image is surrounded by a hyperintense ring. However, tuberculomas in the brain stem can be confounded with astrocytic tumors, especially when no immunological deficiencies or relevant medical history are present. This case report aims to show the diagnosis and process in a patient with pontine tuberculoma and a review of tuberculomas in the pons.

## Case presentation

History and examination

We present the case of a 39-year-old male who was a construction worker for several years, right-handed, who consumed between 190 and 240 mg of alcohol every weekend. There are no additional antecedents of risk or evidence of AIDS. He was not in contact with patients with TB. Four months before hospitalization, the patients showed progressive paraparesis, two weeks before neurological attention that turned into paraplegia. Three days before hospital admission, his quality of life deteriorated for truncal ataxia and quadriplegia (Daniels scored 0/5 in superior limbs and 1/5 in inferior limbs). One week before coming to our hospital, dysarthria and bilateral facial palsy were added to the clinical manifestation. During hospitalization, the patient showed a Glasgow score between 14 and 15. He presented bilateral hypoesthesia to taste on the posterior third of the tongue, severe dysphagia (eventually, he received a nasopharyngeal tube), bilateral hypotonia, and hypotrophy of trapezius and sternocleidomastoid muscles.

Investigations

**Figure 1:**
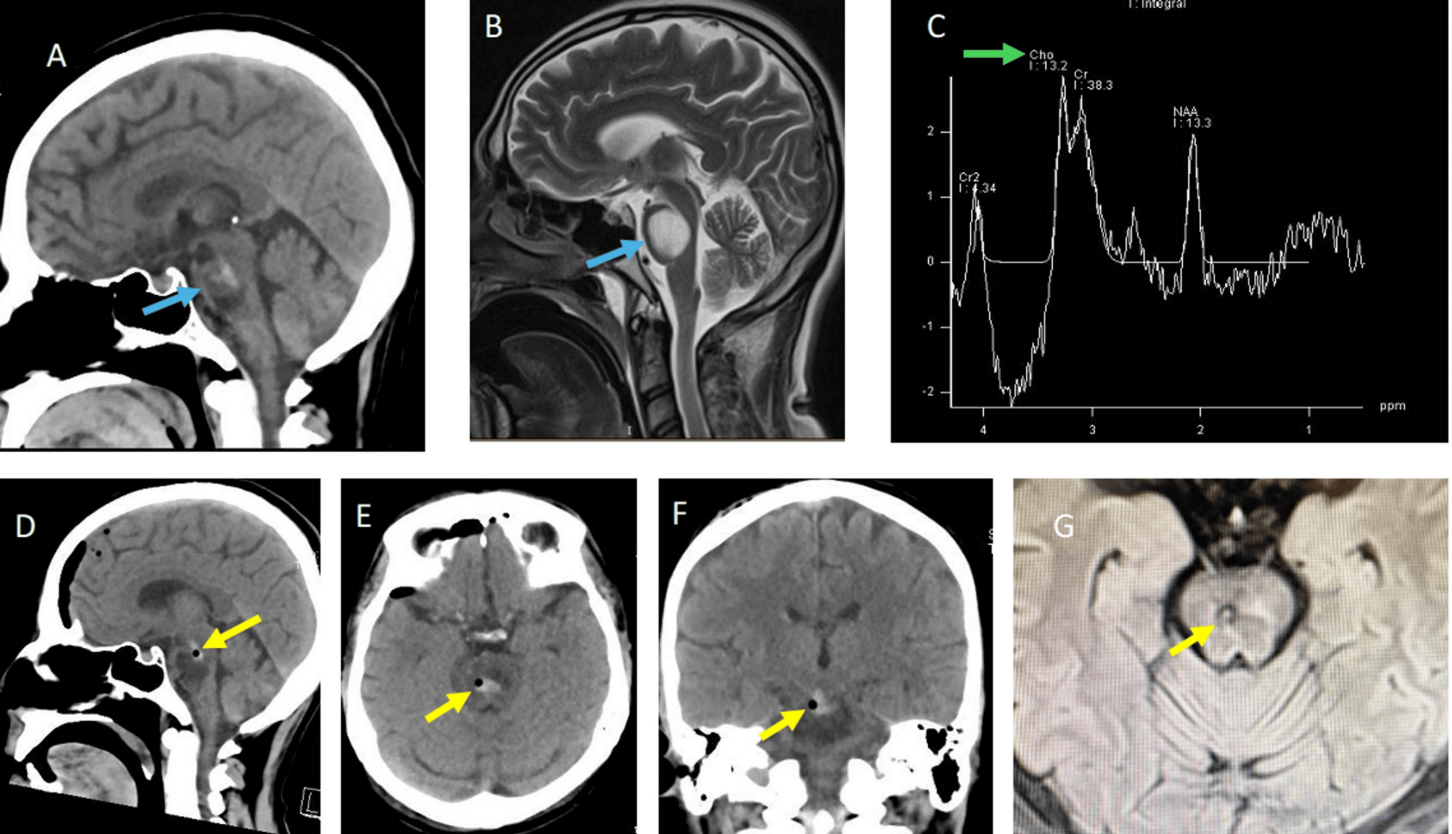
(A) Sagittal images in CT scan and (B) T2 sequence of MRI show ovoidal lesion into the pons (blue arrows). (C) Spectroscopy shows the inversion of choline and the ratio of creatine/N-acetyl-aspartate (green arrow). (D) Sagittal, (E) axial, and (F) coronal images in CT scan follow-up after first biopsy (air bubble is observed in the rim of the lesion) (yellow arrows). (G) Similar images are shown in MRI in fluid attenuated inversion recovery (FLAIR) sequence.

CT scan with contrast showed heterogeneous hypodense and hyperdense images in the pons (Figure 1A). MRI showed a heterogeneous hypointense and isointense image in T1 and T2 sequences. MRI with gadolinium showed hyperintense images in T1 and T2 sequences (Figure 1B). The lesion size was 40 × 22 × 25 mm. Spectroscopy had an inversion of the ratio creatine-choline/N-acetyl-aspartate (Figure 1C). The patient was programmed for stereotactic biopsy to confirm probable astrocytic neoplasia in the pons. Surgery was performed with Zamorano-Dujovny frame (F. L. Fischer, Freiburg, Germany) and Praezis software (inomed, Emmendingen, Germany). Twelve samples were collected and intraoperative analysis was performed. Macroscopic analysis showed white-pink, friable tissue. The sample did not show purulent material. In microscopic analysis, cytological detail of the smear with the presence of capillary vessel proliferation, in addition to glial proliferation with preservation of some neurons.

After surgery, he received intravenous dexamethasone for seven days. The patient had a mild recovery of tetraplegia (Daniels score 3/5) and dysphagia for one week. 

**Figure 2:**
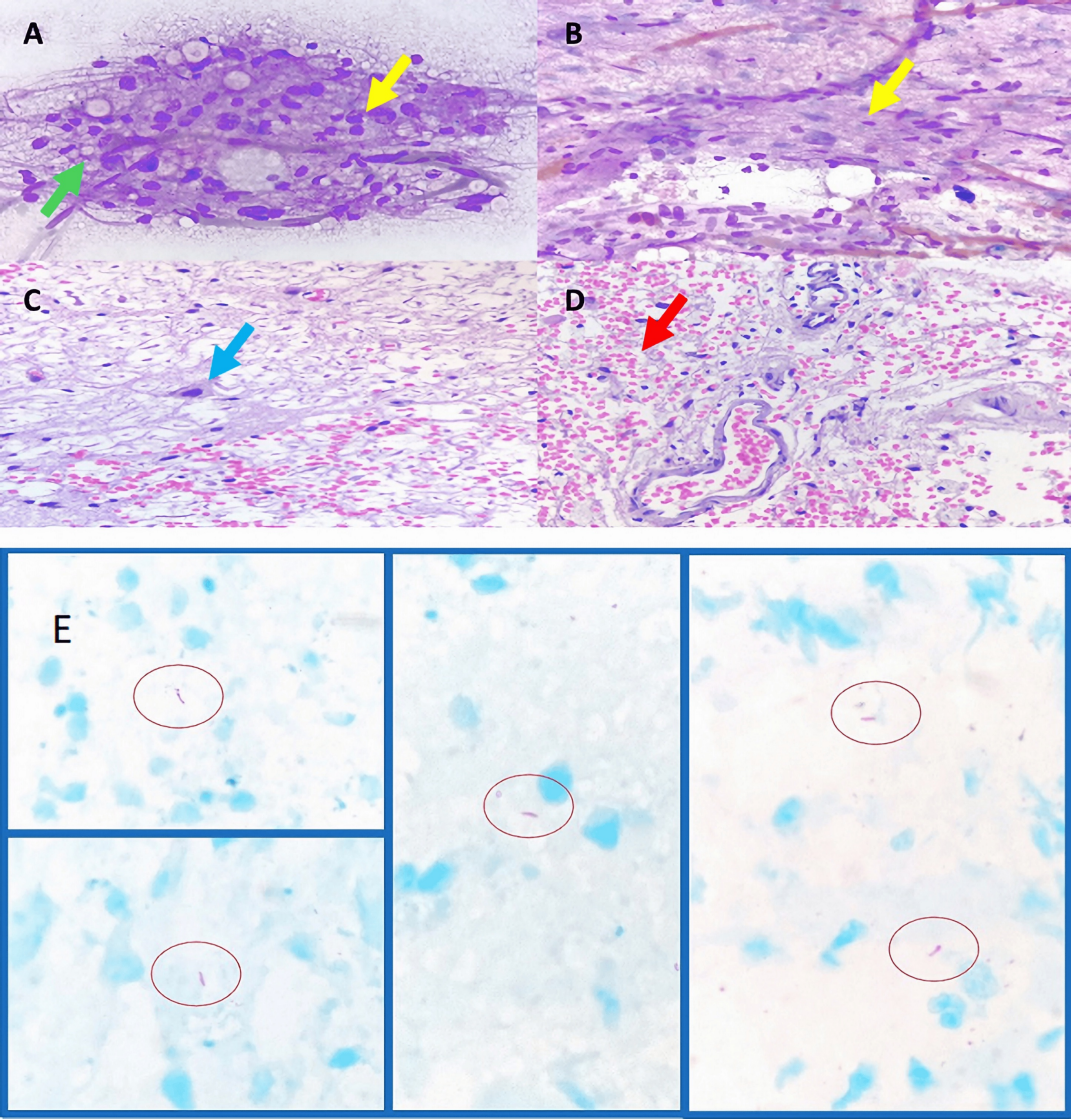
Cytological and histopathological findings. (A, B) Cytological detail of the smear with the presence of capillary vessel proliferation (green arrow), in addition to glial proliferation with preservation of some neurons (yellow arrows) (Hemacolor stain, 400×). (C, D) Cell block with accentuated edema, there is preservation of neuronal bodies and axons (blue arrow), vascular proliferation (red arrow), and erythrocyte extravasation (H&E, 400×). (E) Cell block from the second biopsy performed, five acid-alcohol-resistant bacilli were identified (ellipses) (Ziehl-Neelsen (ZN) stain, 1000×).

However, the biopsy report indicated an ischemic/inflammatory process, inconclusive, with no evidence of neoplasia (Figures 2A-2D).

**Figure 3:**
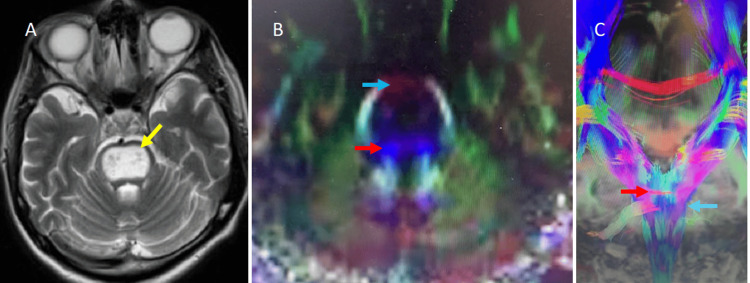
(A) Axial MRI hyperintense image shows a heterogeneous hyperintense lesion in the pons (yellow arrow). (B, C) The axial and coronal images with diffusion tensors of the pons show compression of corticospinal fibers in blue (blue arrows) and transverse pontine fibers in red (red arrows).

Postoperative CT scan showed an adequate biopsy location (Figures 1D-1F). A second MRI with gadolinium was made, but unlike the first MRI, the pons showed an iso-hypointense image surrounded by an enhanced rim (Figure 3A). Tractography in the brain stem showed a displacement of corticospinal tracts into the pons (Figures 3B-3C).

**Figure 4:**
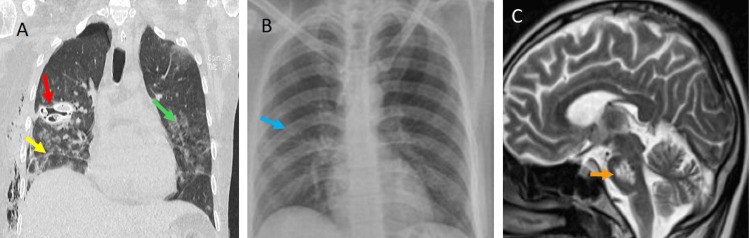
(A) CT scan of the chest shows cavitated lesions (red arrow), cisuritis (yellow arrow), and interstitial infiltrate (green arrow). (B) Three-month follow-up X-ray image with complete recovery (blue arrow). (C) Partial decrement of size lesion in the pons after three months of treatment (orange arrow).

Because of the clinical condition of the patient and lack of specific diagnosis, he was undergoing a second stereotactic biopsy. In this surgery, the samples were taken from the center of the lesion despite the appearance of necrosis. The second biopsy reports mycobacteria associated with reactive glia and macrophages (Figure 2E). The patient developed pneumothorax and hypoxemia forty-eight hours after the last biopsy. A chest CT scan showed several lung cavitation areas in combination with consolidation zones (Figure 4A). Adenosine deaminase test (126.6 U/L) and COVID-19 polymerase chain reaction (PCR) tests were positive. The diagnoses of pulmonary TB and pontine tuberculoma were established.

Evolution

Treatment with streptomycin, isoniazid, and rifampicin was prescribed for at least one year, physical rehabilitation for four weeks, and a hypercaloric diet was started for weight gain. The quadriparesis at three-month follow-up was rated as 4/5 on the MRC score. Six months later, the patient had a normal gait without additional neurological deficit. The X-ray was completely normal (Figure 4B), and the MRI showed a reduction in the size of the pontine lesion (30 × 20 × 20 mm; Figure 4C).

## Discussion

The present work aims to describe a rare lesion in the brainstem exhibiting some clinical and imagological features consistent with astrocytic tumor despite the absence of immunodepression or a history of TB. In the beginning, heterogeneous and hypo/hyperintense MRI images were recorded. In Table 1, there is a list of 10 articles published from 1994 to 2016 with information about tuberculomas in the pons. In 1998, Kauner-Fisher et al. reported a pontine tuberculoma in a Somali 12-year-old child who was diagnosed with astrocytoma in the pons with pulmonary infection [3].

Sugimori et al. published a similar case with a suspicious image in the lungs and a history of TB; with this information, he started the anti-TB treatment and had a good response [4]. Turan Suslu et al. in 2010 reported a case of a 30-year-old woman with headache, seizures, and MRI spectroscopy with a decrement of N-acetyl-aspartate/choline ratio and increment of choline/creatine ratio [5]. Lyons et al. in 2013 showed a TB pontine lesion with partial displacement of cortico-spinal pathways [6]. In 2016, Mora et al. informed about a stereotactic biopsy and drain of abscess by TB in the pons, which had good clinical results [7].

Sadashiva et al. in 2017 reported a series of 14 cases with brain stem tuberculomas [1]. Some common features included a predominance in young adults, with no antecedent of pulmonary infection, a man/woman ratio of 1.5, first attention to neurological syndrome done between four and five months, and more frequent locations being the pons (11 out of 14) (Table 1).

**Table 1:** Eleven cases reports of pontine tuberculomas

Author	Year	N	Sex	Age (years)	Time previous to diagnosis (months)	Clinical features	Comorbidities (immunodeficiency)	Laboratory findings	Image	Location	Stereotactic biopsy	Treatment	Outcome
Knauer-Fischer et al. [3]	1999	1	Male	12	8	Headache, vomit, cough, vertigo, ataxic gait, paresis of the right sixth nerve, diplopia, paraplegia, and hyperreflexia	None	Light increment of lactate dehydrogenase (LDH)	Chest X-ray with TB lesion, MRI T1 isointensity and TA hypointensity, major diameter of 25 mm	Pons	No	Empirical tetratherapy anti-TB, but chemical hepatitis, adjusted to two medicaments	Complete recovery MRI image of 10 mm
Minagar et al. [8]	2000	1	Male	27	3	Headache, weakness, diplopia by left internuclear ophthalmoplegia, nystagmus, right VII nerve palsy	AIDS, pulmonary TB, Pneumocystis carinii pneumonia, pulmonary histoplasmosis, septicemia by Staphylococcus	Cerebrospinal fluid in lumbar puncture was cloudy and xanthochromic, with 915 white blood cells. Glucose 18 mg/dL, protein 611 mg/dL. Adenosine deaminase (ADA) 30 (reference value of 9).	MRI showing a hyperintense lesion in the pontine tegmentum	Pontine tegmentum	No	Isoniazid, rifampin, and ethambutol	Partial recovery
Sugimori et al. [4]	2002	1	Male	66	1	Headache, paresis of left fourth nerve, diplopia, sluggishness in speech, right facial and corporal hemisensory	TB 40 years ago	Leukocytosis 9,950/mm^3^, C-reactive protein 10.1 mg/dL	Small, multiple, calcified nodules in chest X-ray. CT-scan with lesion of 10 mm in diameter, showing hyperdensity. MRI lesion of 15 mm in diameter in T1 heterogeneous image with isointensity and hypointensity, T2 with areas of hyperintensity with gadolinium	Pons	No	Empirical therapy with Isoniazid, rifampin, and ethambutol	Complete recovery MRI image disappeared
van Toorn et al. [9]	2006	1	Female	4	<1	Clumsiness, increased head circumference, lymph nodes in inguinal regions	None	C-reactive protein 9.9 mg/L (0-5 mg/L)	Chest X-ray: opacification of anterior segment of the right upper lobe. Hepatomegaly in USG and CT scan. MRI with T1 lesion hypointense central region with a ring-enhancing	Pontine tegmentum	No	Isoniazid, pyrazinamide, rifampin, and ethambutol, plus corticosteroid	Complete recovery MRI image disappeared
Saxena et al. [10]	2006	1	Male	38	1	Right gaze palsy	None	Elevated erythrocyte sedimentation, white blood count of 18,400/mm^3^, 55% lymphocytes, Mantoux test positive	Chest X-ray: bilateral, apical opacification in lungs. Bilateral hilar lymphadenopathy, MRI with T1 lesion isointense central region with a ring enhancement	Pontine tegmentum in right side	No	Isoniazid, pyrazinamide, rifampin, and ethambutol	Complete recovery, reduction of image in MRI
Winklhofer et al. [11]	2011	1	Female	49	<1	Slowly progredient swelling in her tongue with dysphagia	TB in lungs during childhood. Multiple myeloma at 11 years old. Immunosuppression by cyclophosphamide	Lymphopenia 520/mL, increment of C-reactive protein 135 mg/L	Diffuse reticulonodular opacity in chest X-ray. Disseminated pulmonary and pleural nodules in CT scan. Lesion of 8 mm in diameter, hypointense in T1 without contrast, hyperintense ring with gadolinium.	Pons (left brachium conjunctivum)	No	Empirical tetratherapy anti-TB	Complete recovery MRI image disappeared
Gurjar et al. [12]	2013	1	Male	14	1	Fever, right spastic hemiparesis, left facial paresis, diplopia	None	NA	Globular pontine lesion, hypointense in T1, heterogeneous with hyperintense ring in T2. Spectroscopy with lipid-lactate peak and increased choline/N-acetyl-aspartate (NAA) ratio	Pons and medulla	No, resection by craniectomy	Streptomycin, isoniazid, pyrazinamide, rifampin, and ethambutol	Partial recovery, reduction of image in MRI
Lyons et al. [6]	2013	1	Female	43	1	Headache, room-spinning dizziness, blurred vision, left facial and corporal hemisensory and motor deficit, dysmetria, dysdiadochokinesia, hyperreflexia, Hoffman and Babinski on left side	None	NA	Multiple retroperitoneal, pelvic, and axillary lymph nodes. Apical lungs with granulomatous disease. MRI in T1 hypointense lesion of 29 × 27 × 25 mm with hydrocephalus for the fourth ventricle occupied. T2 with hyperintensity surrounding lesion. Diffusion tensor image with truncation of right fiber tracts	Pons (right side)	No	Biopsy of axillary node. Treatment with ampicillin, isoniazid, pentamidine, and ethambutol	Complete recovery, decrease size image in MRI
Kim et al. [13]	2015	1	Female	76	<1	Orthopnea, pleural effusion, headache, dysarthria	None	Pleural fluid with 907/mm³ white blood cells, 30% lymphocytes, adenosine deaminase in pleural liquid 54.6 IU/L	CT scan of chest showed miliary nodules and centrilobular nodules. MRI hypointense lesion in T1, isointense lesion with hyperintense ring in T2. Edema present.	Pons (left brachium conjunctivum)	No, resection by craniectomy	Isoniazid, pyrazinamide, rifampin, and ethambutol without response at the beginning.	Partial recovery, reduction of image in MRI
Mora et al. [7]	2016	1	Male	49	2	Headache, dizziness, left corporal hemisensory and motor deficit, dysarthria, dysphagia, lateropulsion to right and nystagmus	None	Normal	T1 hyperintense ring with gadolinium	Pons (right side)	Yes	Anti-TB therapy	Complete recovery, decreased size of the image in MRI
Jimenez-Ponce et al.	2024	1	Male	39	4	Tetraplegia, dysphagia, dysarthria, bilateral facial paresis, dysfunction of taste, XI and XII cranial nerves	Alcohol consumption	Adenosine deaminase of 126.6 U/L in second biopsy	Globular pontine lesion, hypointense in T1, heterogeneous with hyperintense ring in T2. Spectroscopy with lipid-lactate peak and increased choline/NAA ratio	Pons	Yes (two biopsies)	Isoniazid, rifampicin, and streptomycin	Partial recovery, reduction of image in MRI
Pooled data	1999-2024	11	Male: 7/ female: 4	37.91 ± 21.37	<1 to 8	Headache, diplopia, dysarthria, hemiparesis, dysphagia	Normal: 7/immunodeficiency: 4	With blood cells increased with abnormal number of lymphocytes, C-reactive protein increased	MRI hypointense lesion in T1, isointense lesion with hyperintense ring in T2. Pulmonary lesion in chest X-ray	Lesion in the pontine tegmentum, brachium conjunctivum, or entire pons	No: 7/craniotomy: 2/stereotactic biopsy: 1	Isoniazid, streptomycin, rifampicin, pyrazinamide, and ethambutol	Complete recovery MRI image disappeared: 7/partial recovery, reduction of image in MRI: 4

This study shares characteristics similar to Sadashiva et al.’s report, such as sex ratio, location, absence of TB history or immunosuppressive disorder, MRI image features, and good clinical response to drug vs. mycobacteria [1]. The small number of stereotactic biopsies is outstanding. Sadashiva et al. report that this procedure is not conclusive.

Different data were the average age (24.7 years vs. 37.9 years, present case), the time to diagnosis in the present study was <1 month vs. Sadashiva et al., with four to five months. In this series of cases, extracranial TB was shown in 50% of patients, contrary to Sadashiva et al. [1]. The spectroscopy was abnormal in eight of 14 Sadashiva et al.’s series and just two of 11 cases of the present review. Tractography showed different wide displacements on adjacent structures depending on the size of the lesion.

Diagnosis of pontine tuberculoma must be suspected, and the physician should analyze the clinical manifestations, laboratory results, and image data. Indeed, a neurological syndrome in the pons (Millard-Gubler, Raymond, and inclusive Marie-Foix syndromes) could be observed with lesions in this anatomical area. Brain tumors can be misdiagnosed instead of TB [3]. In this series, the most frequent symptoms were headache, diplopia, dysphagia, and pyramidal syndrome. MRI is important for recognizing tuberculoma because it offers the main clues [9].

Isolation of etiological agents is very difficult. The biopsy samples do not confirm the diagnosis either, as an expert neuropathologist must study the interpretation of microscopic findings. Even more, additional data, such as immunosuppression or laboratory tests, are useful but they are only available sometimes. Anti-TB treatment is very efficient, and in just two cases, surgical resection was needed [1]. The objectives of stereotactic biopsy are to confirm the etiological agent and analyze the histopathological features.

## Conclusions

The microscopic analysis during the transoperative period of stereotactic biopsy is mandatory. Any case of pons lesion must raise suspicion of TB. The pons is the most frequent location of brain stem tuberculomas; its more frequent symptoms are headache, diplopia, upper motor neuron syndrome, cerebellar syndrome, and nerves VI and VII dysfunction. MRI images show an iso/hypointensity pattern in the T1 sequence and hypointensity with a rim of hyperintensity in the T2 sequence. Spectroscopy is abnormal in lipids and the ratio choline/N-acetyl-aspartate. Brain biopsy is not conclusive in many cases. Immunosuppression history and TB antecedent are not clearly defined. Specific medication treatment is very successful.

In this case report, the delay to perform the differential diagnosis did not produce permanent side effects, but result in a retardation of adequate treatment.
